# Characteristics of the Beef Cheek Meat-Based Sausage Added with Snakehead (*Channa striata*) Gelatin

**DOI:** 10.1155/2023/6877904

**Published:** 2023-02-01

**Authors:** Rosmawati, Abu Bakar Tawali, Muhammad Irfan Said, Sri Fatmah Sari, Lely Okmawaty Anwar, Iin Nurdianty Nurdin, Anwar Said, Ary Tamtama, Fuji Astuty Auza, Wahidu Zzaman, Mohammad Halim Jeinie, Mohd Nazri Abdul Rahman, Nurul Huda

**Affiliations:** ^1^Study Program of Fishery Products Technology, Faculty of Fisheries and Marine Sciences, University of Muhammadiyah Kendari, Jalan K. H. Ahmad Dahlan No. 10, Kendari 93117, Indonesia; ^2^Faculty of Agriculture Science, Laboratory of Food Science and Technology, Hasanuddin University, Jalan Perintis Kemerdekaan, South Sulawesi, Makassar 90245, Indonesia; ^3^Faculty of Animal Science, Laboratory of Animal Byproduct Processing Technology Hasanuddin University, Jalan Perintis Kemerdekaan, South Sulawesi, Makassar 90245, Indonesia; ^4^Faculty of Animal Science, Halu Oleo University, Jalan H.E.A Mokodompit Kampus Anduonohu, Kendari, 93231 Southeast Sulawesi, Indonesia; ^5^Department of Food Engineering and Tea Technology, Shahjalal University of Science and Technology, Sylhet 3114, Bangladesh; ^6^Faculty of Food Science and Nutrition, Universiti Malaysia Sabah, Jalan UMS, Kota Kinabalu, 88400 Sabah, Malaysia; ^7^Faculty of Sustainable Agriculture, Universiti Malaysia Sabah, 90509 Sandakan, Sabah, Malaysia

## Abstract

This study is aimed at determining the functional effect of snakehead fish gelatin as a binder on the characteristics and shelf life of beef cheek-based emulsion sausage compared with bovine commercial gelatin. The level of snakehead fish gelatin used was 0%, 1%, 2%, and 3%, while that of bovine commercial gelatin was 2% with a storage time of 0 to 28 days in the refrigerator (4 ± 2°C). Emulsion stability, cooking loss, proximate composition, texture profile, and microstructure of sausage were initially determined before storage; then, observations were made every seven days to determine the shelf life of sausages based on pH, antioxidant activity, and TBA reactivity. Characteristics such as emulsion stability, proximate composition, and texture profile were influenced by the treatment (*p* < 0.05). The gelatin level significantly affected the water holding capacity of sausages (*p* < 0.05), but the storage time did not (*p* > 0.05). On the other hand, the pH, TBA reactivity, and antioxidant activity of sausages were not only affected by gelatin level (*p* < 0.05) but also by storage time (*p* < 0.05). The sausage microstructure confirms the use of 2% snakehead fish gelatin to make sausages with properties similar to 2% commercial bovine gelatin. The byproduct of the snakehead fish industry can be used as a gelatin alternative to produce sausages. This gelatin has the potential as a binder, which can functionally improve sausage characteristics. This effectiveness can boost the water holding capacity of sausages, although it has not been effective in inhibiting fat oxidation which causes an increase in malonaldehyde levels.

## 1. Introduction

Future food sustainability has a challenge and an opportunity with the problem of global change and the momentum of the fourth industrial revolution, both of which must be quickly addressed. One of the points that are of particular focus to be resolved is generating new food product trends and the use of food byproducts [1]. Currently, animal byproducts have been designated as an alternative future food source to complement the needs of the main source of food. In reality, a large population should be proportional to the demand for adequate food. Thus, the byproduct has the opportunity to fulfill these needs. As a consideration, byproducts of animal origin still contain potential nutritional value to be developed into products that are acceptable to consumers [2].

One of the byproducts of beef livestock that is used as well as carcass meat is beef cheek meat. Beef cheek meat has the same characteristics as carcass muscle even though it is classified as noncarcass muscle. The characteristics of beef cheek meat resemble abdominal muscle meat [3] and have a moderate water holding capacity [4] with an average composition of 75.75% moisture, 22.25% protein, 0.31% fat, and carbohydrates and ash < 1% [5]. Because it resembles skeletal meat, beef cheek meat can be diversified into various processed products. Several regions in Indonesia use this meat for traditional meat-based dishes such as soup and soto.

Fish gelatin is a byproduct that is mostly produced from fish skin and bones left over from the fishery processing industry. Gelatin is a potential derivative product of collagen protein and can be applied in various industries, both food and nonfood industries [6–8]. The use of gelatin in the processing of emulsion products such as surimi [9, 10] and sausages has been widely practiced [11–15]. This study will try to observe the effect of adding snakehead fish gelatin to sausage-based beef cheek meat. Rosmawati et al. [16] revealed that the snakehead fish's skin had a gel strength ranging from 108.00 ± 21.40 to 278.37 ± 22.28 and its internal gel strength ranged from 41.37 ± 7.42 to 239.53 ± 20.69, treated as the foundation for the use of snakehead fish gelatin as additives that function as binders and emulsifiers at the same time.

Sausage is a processed food that continues to innovate in its composition and formulation. The use of several emulsifiers, both based on polysaccharides [17] and protein [9, 13, 14], is still a suitable alternative for sausage products. The nature of the emulsion in sausages is two phases in the dough with polar and nonpolar charges which can affect each other if the intermediate binder works well and is well dispersed in the liquid and fat added to the dough. The ability of flat meat to maintain water holding capacity will decrease if the composition of ingredients in the sausage formulation increases, followed by the properties of other ingredients with characteristics that are not as good as meat in binding water and fat. Therefore, it is necessary to consider adding a binder that also functions as an emulsifier to maintain sausage stability during processing and storage, as Tan et al. [18] explained that the physical stability of emulsions in water could be formed by gelatin. Snakehead fish gelatin is intended as a binder that can increase the characteristics and shelf life of emulsified sausages made from beef cheek meat.

The purpose of this study was to compare snakehead fish gelatin to commercial gelatin in order to ascertain the functional impact of snakehead fish gelatin as a binder on the characteristics and shelf life of emulsified sausages made from beef cheek meat.

## 2. Materials and Methods

### 2.1. Materials

Beef cheek meat and back fat were obtained from the Tamangapa Slaughterhouse, Makassar, South Sulawesi, Indonesia. Snakehead fish gelatin used was the result of a partial hydrolysis process from a previous study [16] through pretreatment with calcium hydroxide, treatment process with citric acid, and extraction at 60°C for 12 hours. Snakehead fish gelatin sausage formula ingredients include soy protein isolate (Marksoy 90, Shandong Crown Soya Protein Co. Ltd., China), tapioca flour (Rose Brand), milk flour (PT. Nestle Indonesia), seasoning (Frankfurter Combi Forte, PT. Markaindo Selaras), pepper powder (Cap Koepoe Koepoe, PT. Gunacipta Multirasa), garlic powder (Cap Koepoe Koepoe, PT. Gunacipta Multirasa), potassium nitrite (Cap Koepoe Koepoe, PT. Gunacipta Multirasa), ice, salt, sodium tripolyphosphate, collagen casings (18.45 mm) (Devro, PT. Markaindo Selaras), and the commercial gelatin (gelatin of bone bovine, 150 g gel strength, Green Valley, Global Capsule Ltd., Dhaka 1000, Bangladesh). Chemicals for experimental purposes were all of analytical grade.

### 2.2. Methods

#### 2.2.1. Sausage Preparation

Beef cheek meat was cleaned and then stored at 4 ± 2°C for 24 hours. Before processing, the connective tissue of the beef cheek meat was removed. The sausage was made based on the formulation of 50% beef cheek meat, 20% ice water, 20% fat, and 10% nonmeat ingredients (soy protein isolate, tapioca flour, milk flour, seasoning, salt, sodium tripolyphosphate, and potassium nitrite). The percentage of snakehead fish gelatin (SFG) in the total material used was 0%, 1.0%, 2.0%, 3.0%, and 2.0% commercial bovine gelatin (BCG) as a comparison. Sausage formulation and manufacturing method refers to Mohan [19] with some changes and conversions. Sausage ingredients were ground using a meat processor (Miyako, CH-501 PF AP) to form the emulsion. The dough that had formed the emulsion was partly used for testing the stability of the emulsion and partly for making sausages. The dough was put in a casing and cooked in water at 80 ± 3°C until it floats (about 20 minutes). The cooked sausage was allowed to be at room temperature, for further analysis. To determine the shelf life of the sausages, it was stored for 28 days in a refrigerator at 4 ± 2°C. Analysis was made on days 0, 7, 14, 21, and 28 of the storage periods.

#### 2.2.2. Emulsion Stability

The determination of sausage emulsion stability was performed as described by Mohamed et al. [20]. A sample of about 30 g was weighed and placed in a 50 mL centrifuge disposal tube and centrifuged at a speed of 2,800 × g at 4°C for 15 minutes with a high-speed refrigerated microcentrifuge (Tomy Mx-305), to remove air bubbles. The tube was then heated at 70°C for 30 minutes in a water bath (Memmert W350, Germany) and allowed to stay at room temperature. The tube was centrifuged again at a speed of 2,800 × g at 4°C for 15 minutes. The supernatant (S) contained in the tube was poured into a porcelain crucible. The released supernatant was weighed (RS) and dried in an oven (Shimadzu) at 105°C for 16 hours, to obtain the volume of released water (RW). The porcelain crucible was weighed again to determine the weight of fat released (FR). The percentage of fat released (FR) was determined as the difference between the percentages of S and RW.

(1) $$\begin{array}{c} \%\text{S}=\frac{\text{RS}}{\text{Sample weight}}\times 100,\\ \%\text{RW}=\left[\left(\text{Porcelain crucible weight}+\text{S}\right)-\left(\text{Dry weight of porcelain crucible}\right)\right]\times 100. \end{array}$$

#### 2.2.3. Proximate Analysis

Sausage samples were analyzed proximately according to the guidelines of the AOAC [21]. Moisture content was determined using the gravimetric method. The crude protein determination method using the Kjeldahl method was based on a conversion factor of 6.55 × *N*. The Soxhlet method was used to quantify the fat content and the ash content by incineration at 550°C for 16 hours.

#### 2.2.4. Cooking Loss

The measurement of sausage cooking loss refers to the method of Asmaa et al. [22]. Sausages were first weighed to determine the initial weight. After cooking, it was allowed to adjust to room temperature and then reweighed to calculate the percentage of released water. A 40 g sample was put in a plastic clip and then cooked in a water bath (Memmert W350, Germany) at 70°C for 30 minutes. The cooked sample was removed from the plastic and left for about 1 hour so that the water that was still attached to the outside of the sample had evaporated.

(2) $$\begin{array}{c} \text{Cooking loss }\left(\%\right)=\frac{\text{Initial weight before cooking }\left(\text{g}\right)-\text{Weight of sausage after cooking }\left(\text{g}\right)}{\text{Initial weight before cooking }\left(\text{g}\right)}\times 100. \end{array}$$

#### 2.2.5. Profile Texture

Sausage texture was measured using the TAXT2i Texture Analyzer (Stable Micro System, UK). Determination of the sausage shear force uses a probe knife (WDP probe) with a distance of 25 mm from the sample. Hardness, compactness, and elasticity were determined by placing the sample under a blunt probe (SMS 35 probe) and then pressing it to 50% for 5 seconds. The whole measurement process requires a speed of 2 mm/s with a maximum load of 25 kg [23].

#### 2.2.6. Sausage Microstructure

Microstructural observations was performed using a scanning electron microscope (SEM, Hitachi SU3500), according to the instructions of Rombe et al. [24]. The sample was lyophilized first; next, it was attached to gold metal, which had been coated with carbon glue in a vacuum plasma tube device that produces microwaves (magnetron sputtering devices) equipped with a vacuum pump. Towards the end of the 20-minute vacuum process, gold metal leaped towards the sample. The coated sample was placed in its position in the electron microscope, and any instances of electrons shooting towards the sample were recorded on the monitor.

#### 2.2.7. Water Holding Capacity

The water holding capacity of the sausage was measured using the filter paper press method [5]. About 300 mg of the sausage was placed on filter paper and pressed on two plates weighing 35 kg/cm^2^. After 5 minutes, the area covered by the wet sample and the surrounding area was marked and measured with a planimeter. The wet area was the area of water absorption on the filter paper after being clamped for 5 minutes minus the area covered by the meat sample. To determine the effect of sausage storage on the ability of sausages to water binding, measurements of water content (mg H_2_O) were carried out at 0 to 28 days and were calculated by the formula as follows:

(3) $$\begin{array}{c} \text{mg }{\text{H}}_{2}\text{O}=\frac{\text{Wet area }\left({\text{cm}}^{2}\right)}{0.0949}-8.0. \end{array}$$

#### 2.2.8. The pH of Sausage during Storage

The sausage pH measurement was carried out every seven days of storage starting from day 0 to day 28. The pH measurement method was referred to as the method of AOAC [21]. Before use, a buffer of pH 4 and pH 7 was used to calibrate pH meter. The pH measurement was carried out by inserting a special electrode into the sausage.

#### 2.2.9. TBARS (Thiobarbituric Acid Reactivity Assay)

The method for analyzing the TBARS value of sausages is described by Tarladgis et al. [25]. A total of 10 g of sample was weighed and crushed together with 50 mL of deionized water with a hand blender for 2 minutes. Before the sample solution was distilled, 47.5 mL of aquabides and 2.5 mL of 4 M HCl were first added. The distillation process was carried out by heating for 10 minutes to obtain about 50 mL of distillate liquid. The distillate obtained was homogenized by vortex (IKA Labortechnik VF2, Germany) and then put in a 5 mL tube. A total of 5 mL of TBARS solution was added to the tube and then heated at 80°C for 30 minutes in a water bath (Memmert W350, Germany). The samples were allowed to cool and then incubated for 6 days in the dark at room temperature. Blanks were made using 5 mL of aquabides added with 5 mL of TBARS solution. The absorbance value (D) of the sample was measured using a spectrophotometer (Shimadzu UV-1800) at a wavelength of 528 nm with a blank solution as the starting point. The sample solution and the blank were put in a cuvette with a diameter of 1 cm. The level of TBARS was expressed in mg malonaldehyde/kg sample.

#### 2.2.10. Antioxidant Activity of Sausage

Measurement of antioxidant activity refers to the DPPH method [26]. About 0.008 g of DPPH was weighed and then diluted into 50 mL of methanol. 0% absorbance was obtained from dilution of DPPH at several concentrations. The dilution was carried out by adding DPPH to 9 mL of methanol with each concentration of 50 *μ*L, 60 *μ*L, 70 *μ*L, 80 *μ*L, 90 *μ*L, and 100 *μ*L. The absorbance of the solution was measured using a UV-VIS spectrophotometer (Thermo Fisher Scientific 4001/4, Genesys VF2, Germany) at a wavelength of 515 nm. Sausage sample 1 g was diluted into 9 mL methanol and homogenized by vortex (IKA Labortechnik VF2, Germany). The dilution was carried out from 10^−1^ to 10^−5^. Each dilution was tested with as much as 0.2 mL of sample solution into a test tube and added 3.8 mL of DPPH solution and 0.2 mL of methanol. The sample mixture was homogenized by vortex (IKA Labortechnik VF2) and allowed to stand for 60 minutes in a dark room. The absorption of the solution was measured using a UV-VIS spectrophotometer at a wavelength of 515 nm. The amount of antioxidant activity is calculated by the following formula:

(4) $$\begin{array}{c} \text{DPPH }\left(\%\right)=\frac{A_{\text{DPPH}}-A_{\text{Sample}}}{A_{\text{DPPH}}}\times 100, \end{array}$$

where *A*_DPPH_ is the absorbance of DPPH and *A*_Sample_ is the absorbance of the sample.

#### 2.2.11. Data Analysis

Data analysis was conducted using SPSS software (IBM Corporation, US). Each treatment was repeated 3 times, and the average data were expressed as mean ± SD. Data showing a significant effect at *p* < 0.05 will be continued with Duncan's test. Scanning electron microscope analysis was tested descriptively and qualitatively.

## 3. Results and Discussion

### 3.1. Sausage Emulsion Stability

The results in Table 1 show that the addition of gelatin had a significant effect (*p* < 0.05) on the stability of the sausage emulsion, which was expressed as total liquid/supernatant released (S), released water (RW), and fat released (FR). Further analysis showed that the percentage of supernatant with the addition of 3% SFG was the best among all treatments but did not differ from the 2% SFG and 2% BCG treatments (*p* > 0.05), while the 2% SFG, 2% BCG, and 1% SFG treatments were significant to each other the other way around (*p* < 0.05). Less supernatant (S) was produced when the SFG level was increased, indicating that gelatin was more effective at binding water and fat.

Further investigation revealed that the RW percentage of sausages varied (*p* < 0.05) among treatments. However, between the treatments of 3% SFG, 2% SFG, 2% BCG, and 1% SFG was no significant difference (*p* > 0.05), and such was the case between treatments of 2% BCG, 1% SFG, and 0% (*p* > 0.05). The level of gelatin to the FR of sausage also indicated a difference between treatments (*p* < 0.05). The 3% SFG was significantly different from the other treatments (*p* < 0.05), while treatments of the 0%, 1% SFG, 2% SFG, and 2% BCG had similar values (*p* > 0.05). The addition of up to 3% SFG showed the most effective increase in the amount of fat that can be bound by protein during the emulsification process.

The sausage emulsion was more stable having the higher the SFG content. RW and FR percentages treated with SFG revealed no differences with BCG at the 2% level, which means that the effectiveness of SFG was not much different from commercial gelatin in influencing sausage emulsion stability. The emulsification process for the occurrence of emulsion stability follows the emulsion theory that fat was stabilized in meat dough to form a protein surface layer/interfacial protein film around small fat globules [27]. In addition, this film acts as an interface between the aqueous phase and the fat and prevents the formation of big particles by water and fat (coalescing).

In general, emulsion stability was not solely influenced by gelatin. Santhi et al. [28] stated that emulsion stability can be influenced by the type and content of fat, added water content, fiber type, additives, salt concentration, processing method, and others. Emulsion stability in this study was closely related to the capability of gelatin to bind to water and fat, which was characterized by a significant difference between the treatment with and without the addition of gelatin. Barbut [27] emphasizes that the main parameter that should be minimized in the cooking process of meat products was the release of water and fat. Thus, the lower RW and FR with higher levels of gelatin indicate the effectiveness of gelatin in binding water and fat.

Emulsion stability was crucial for producing sausages with compact qualities. The addition of gelatin as a binder and emulsifier was meant to facilitate the binding of protein to the additional water and fat in sausage dough [4]. According to Feiner [29], the purpose of cooking in sausage making was to emulsify fat, bind water, and at the same time deactivate it when adding water along with the addition of active protein. Gelatin was a hydrocolloid that has an active protein. The activation of this protein was largely determined by the arrangement of some active side-chain amino acids. As stated by Ayadi et al. [30], protein has surface-active substances on both sides that were hydrophilic and hydrophobic. This function can help improve product characteristics [31–33].

### 3.2. Cooking Loss

There was a significant effect (*p* < 0.05) of the addition level of gelatin on the sausage cooking loss. Nevertheless, the addition of gelatin levels of 1% to 3% SFG and 2% BCG showed no difference (*p* > 0.05), although as the gelatin level increased, cooking loss tended to decrease. Cooking losses based on different gelatin levels are presented in Table 1. As the gelatin level increases, the sausage cooking loss tends to decrease. Pereira et al. [4] confirmed that emulsifiers with higher protein content can help improve emulsion stability during cooking. Gelatin is a hydrocolloid that also acts as an additive, water, and fat binder. Gelatin contains high protein levels with almost complete amino acids (except cysteine and tryptophan). The amino acids in gelatin played a role in the incidence of protein binding to fat and water. The hydrophilic and hydrophobic amino acids in sausage dough were responsible for the emulsification that occurred. These amino acids were active proteins that work to bind water on the hydrophilic side and bind fat on the hydrophobic side. Feiner [29] reported that the addition of protein can work actively by inhibiting water mobilization and emulsifying added fat and stabilizing water and fat in a three-dimensional matrix.

Sausage shrinkage occurs due to the released water and fat that was not bound in the dough matrix during the boiling process. When boiling, either water, fat, or gel solution (which may be formed) mixed with other ingredients in the form of a liquid supernatant can seep out causing shrinkage, the more water/fat/gel that was released, the cooking loss will increase [3]. The lower the cooking loss, the higher the sausage yield, and this is an important part of food production because it is related to the efficiency of the use of ingredients in food processing [34, 35].

### 3.3. Proximate Analysis of Sausage

The characteristics of sausage-based beef cheek meat with the addition of SFG at the level of 1%, 2%, and 3% compared to the BCG level of 2% are presented in Table 2. The results of the analysis show that the addition of levels of SFG and BCG affects the moisture content of sausages (*p* < 0.05); however, it did not affect the content of protein, fat, and ash of sausages (*p* > 0.05). No significant differences in sausage moisture content among the treatments 0%, 1% SFG, and 2% BCG were observed (*p* > 0.05), as well as between treatments 1% SFG and 2% BCG, 2% SFG, and 3% SFG (*p* > 0.05).

Beef cheek meat-based sausages treated with different gelatin levels characterized by the moisture content tended to increase along with the rise in the gelatin level, from 60.43 ± 1.73 to 63.95 ± 1.07%. As indicated by Lee and Chin [31], the addition of gelatin up to 1.5% did not affect the moisture content of sausages, but on the contrary, according to the results of Yeo et al. [33], there was a change in the moisture content of sausages as the gelatin concentration increased. The difference in sausage moisture content is associated with the capability of the dough to bind water, and this depends on the type of gelatin with its hydrophilic properties and ability to form gel [29] and the concentration and formulation used [31–33]. The function of gelatin is to bind the excess free water contained in the dough so that the moisture content that will be released during the cooking process is relatively low compared to sausages without the addition of gelatin (0%). Research by Schilling et al. [36] in meat products that pork collagen was added showed a significant reduction in moisture content compared to 0%s.

The addition of gelatin up to 3% SFG and 2% BCG did not affect the protein, fat, and ash content of sausages as shown in Table 2. The range of protein content of beef cheek meat-based sausages was 17.48 ± 0.78% to 19.01 ± 0.67%. The percentage of sausage protein did not differ between treatments (*p* > 0.05), but the addition of gelatin tends to increase the protein content of sausages even though the amount was small. This change in protein content may be due to the contribution of gelatin as a high protein source. The observations of Souissi et al. [32] also showed an increase in sausage protein content from 17.58 ± 0.92% to 20.56 ± 1.07% with the addition of gelatin up to 1.5%. Different levels and types of gelatin can affect the protein content of the sausage produced [4], where the protein content of gelatin varies, depending on the source of the raw material [37].

The fat content of sausage-based beef cheek meat tends to be low, around 2.41 ± 0.36% to 2.69 ± 0.14%. The low-fat content of sausages was related to the amount of fat added and the fat content of the raw material itself, where beef cheek meat has a fat content of <1% [5]. Overall, sausage fat content showed a high value in sausage emulsion treated with SFG 3%, SFG 2%, and BCG 2%, compared to the 0% and SFG 1%. There may be a correlation between fat content and gelatin's capacity to bind fat better at higher gelatin concentrations. Some fats cannot bind completely to proteins in sausages with low gelatin levels, resulting in the breakdown of fat with water during cooking. Fats that are just loosely or not at all bound by protein will become free in the dough and can be easily released into the boiling water. The addition of gelatin as an additive can affect the characteristics of sausages, although this effect depends on the type of raw material used [4, 38].

The ash content of sausage-based beef cheek meat was in the range of 1.86 ± 0.27% to 2.07 ± 0.18%, indicating that the higher the SFG level, the lower the ash content. The ash content of sausages treated with 2% BCG showed a relatively high ash content compared to the ash content of SFG sausages with the same level. The authors suggest that the ash content was more influenced by the addition of gelatin although the effect was not significant.

### 3.4. Sausage Texture Profile

The texture is one of the important attributes and considered both in food ingredients and in the final product. The data analyzed in Table 3 shows that there was an effect (*p* < 0.05) of gelatin level on the hardness, cohesiveness, elasticity, and shear force of sausages.

Further analysis showed that sausage hardness was influenced by gelatin level (*p* < 0.05). The lowest level of hardness was indicated by the 0% treatment; contrary, the 2% BCG treatment represented the highest level of hardness in sausages. There was no statistically significant difference between the 3% SFG and 2% BCG treatments (*p* > 0.05), and there was also no difference among the 3% SFG, 2% SFG, 1% SFG, or 0% treatments on hardness (*p* > 0.05). However, the higher the SFG level, the higher the sausage hardness level, and it seems that 2% BCG gives a better sausage hardness level than 3% SFG. This may be attributed to the better gel strength and hydrophobic properties of BCG than SFG.

According to some studies on the effects of adding gelatin to sausages' hardness characteristics, the availability of water and fat as well as gelatin's ability to act as a binder and blend with other ingredients to create a homogeneous product texture might be related to the increased hardness of sausages at high gelatin levels [11, 14, 39]. The level of hardness of sausage products was inseparable from the type of raw material in the formulation [40, 41]. The hardness level in the 0% sausage was about 3012.90 ± 146.89 g force, lower than that in the fish surimi sausage reported by Santana et al. [40], which was 4.15 ± 0.02 kg force, and higher than that in the chicken sausage reported by Ch'ng et al. [11], which was 230.7 ± 3.74 g force. This indicates that the raw material can affect the level of hardness of the sausage.

Beef cheek meat is a type of meat with relatively moderate water binding capacity, very low-fat content, and relatively high shear force value (Table 3) and visually shows a large amount of connective tissue. At the same time, during heating, there is damage to the connective tissue; however, the extracted connective tissue causes the formation of a gel that melts with water and then binds with free fat and myofibrillar proteins in the dough, causing water and fat to be prevented from escaping from the sausage dough. The trapping of water in the dough was thought to cause the relative hardness of the sausage to increase as the gelatin level increased. This has been studied by Ayadi et al. [30] on the texture of turkey meat and showed that the protein content, protein source, differences in the ratio of fat and water, and the ratio of protein and water affect the texture characteristics of the resulting product. The level of sausage hardness in the study varied from 3012.90 ± 146.89 g force to 4302.70 ± 495.25 g force, depending on the level and type of gelatin.

In further analysis, the cohesiveness of sausage showed a difference among treatments (*p* < 0.05). The addition of 2% BCG showed a higher level of sausage cohesiveness compared to other treatments (*p* < 0.05). Cohesiveness is a measure of the degree of difficulty in destroying internal structures, as Chorbadzhiev et al. [39] mentioned that this texture profile is responsible for sausage integrity. The cohesive power of sausages in different treatments showed values ranging from 0.4 to 0.6. The closer to 1, the better the product integrity. The addition of different SFG levels did not give a different cohesive value from the 0%, although the cohesive level of the 0% treatment was lower than all the SFG treatments. The addition of 2% BCG indicates a better cohesive level among all treatments, and the lower the cohesive value indicates the disintegration of the resulting product, which according to Resurreccion [42] was related to low-quality meat products or the occurrence of structural defects. The addition of a binder such as gelatin can help improve the texture of the sausage, due to an increase in water retention. Sousa et al. [43] explained that the addition of collagen will chemically retain water content through the protein matrix and cause swelling due to contact with water.

The addition of gelatin to sausage elasticity showed the same characteristics between 3% SFG, 2% BCG, and 2% SFG (*p* > 0.05), as well as treatment of 2% SFG, 1% SFG, and 0% (*p* > 0.05). The elasticity of sausage at different gelatin levels showed a value that tended to increase with the gelatin level compared to the 0%, and between 3% SFG treatments, the elasticity level was relatively higher than the 2% BCG treatment. Elasticity is a rheological characteristic that describes the resistance of the product to detachment or rupture due to compressive forces, where the product can return to its original condition when the pressure was removed. This condition is strongly influenced by water retention due to the added binder, as observed by Prestes et al. [44] in adding hydrolyzed collagen to cooked ham products and producing products with higher resistance to compression. The higher the elasticity value, the better the level of texture stability.

The higher the gelatin content, the lower the sausage shearing force, but statistically, the treatments of 3% SFG, 2% BCG, 2% SFG, and 1% SFG have similar values (*p* > 0.05) and different from the 0% (*p* < 0.05). The highest sausage shear force was observed in the treatment without the addition of gelatin and the lowest at 3% SFG. Similar finding was reported by Prestes et al. [44] on the shear force of cooked ham, which had a higher slicing power than those treated with collagen hydrolysate. The higher the sausage level, the lower the sausage shear force value.

The slicing ability of beef cheek meat-based sausages may be connected to the raw meat utilized, as beef cheek meat has relatively difficult qualities due to the thickening of its dense connective tissue. The higher the gelatin content, the weaker the slicing ability of the sausage. In addition to the raw material factor, slicing power can be related to cooking loss. Sausages treated with gelatin will tend to be more tender than those without gelatin because gelatin could bind to water. The added water could cause the sausage's retention of water to be high. Similarly, the use of gelatin results in binding to the fat, which can also give the finished product a softness. The combination of high moisture content with fat that blends more perfectly in the product will give it better properties. Otherwise, in the 0% treatment, where both water and fat were released freely from the product matrix during the heating process, the sausage product was relatively less tender and was characterized by a high cooking loss.

### 3.5. Microstructure of the Sausage

**Figure 1:**
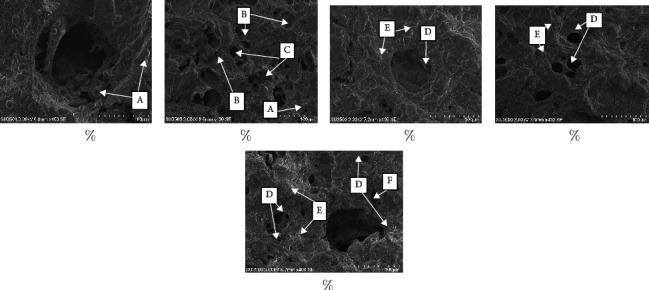
Microstructural profile of sausages at different levels (magnification ×400). (a) 0%; (b) 1% SFG; (c) 2% SFG; (d) 2% BCG; (e) 3% SFG; A = microorganism; B = semicircular hole as a place for unbound fat deposition; C = hole that resembles a pore due to the presence of free water; D = gelatin solution; E = meat fiber; F = cracked sausage surface.

The microstructure of sausages added with different levels of SFG is shown in Figure 1. Sausages emulsified with different levels of gelatin appear to have relatively different structures; according to Parés et al. [45], it was difficult to correlate the appearance of sausage structure with texture. However, the micrograph of the sausage surface for each treatment shows the effect of different sausage formulations and is useful in visually confirming the optimum treatment.

The sausage microstructure shown in Figure 1 presents a clear difference in the cross-section of the sausage surface. Figures 1(a) and 1(b) show the sausage matrix structure which is looser than the sausage matrix structure in Figure 1(c). The loose structure causes the sausage to be less compact, while Figures 1(d) and 1(e) show a more compact sausage structure. The relatively loose structure of the sausage matrix (Figure 1(a)) was due to the inability of water-protein-fat to bind to each other. Due to the water that was released when the heating method was used, this circumstance results in the sausage experiencing syneresis [45]. Sausage micrographs imply the accumulation of water and oil molecules around the cross-sectional surface of the sausage so that the surface layer looks like oil molecules (Figure 1(a)-A). This will have implications for the more rapid deterioration of sausage quality during storage. The addition of 1% SFG (Figure 1(b)) seems to have helped the water-protein-fat pooling, although not yet optimal, which was characterized by the presence of pores as an effect of unbound water (Figure 1(b)-C) and is semicircular as fat deposits that are not bound to the water and protein components (Figure 1(b)-B).

The addition of 2% SFG (Figure 1(c)) showed that the cross-sectional structure of sausages was better than 0% and 1% SFG, where the water-protein-fat binding made the sausage matrix structure look more cohesive. In contrast to the addition of 2% BCG and 3% SFG (Figures 1(d) and 1(e)), the sausage surface appears very tight but produces greater tensile strength, so this condition results in a sausage structure that tends to crack (Figure 1(e)-F). Processing such as grinding will cause damage to the structure of the material, especially the myofibrillar structure of the meat. This myofibrillar damage causes water retention. The addition of fat and water to form an emulsion system in the dough with a damaged myofibril structure character will produce a broken and less compact texture after the heating process occurs. The addition of a binder in the formulation can help the process of unification between myofibrillar protein, fat, and water, thereby contributing to a more compact sausage [46**]**.

### 3.6. Water Holding Capacity of Sausage

**Figure 2:**
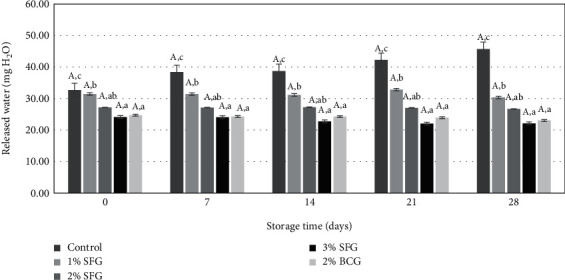
The addition of gelatin level and storage time to the released water (mg H_2_O) of sausage. Different uppercase letters indicate differences in storage time (*p* < 0.05), and lowercase letters indicate differences with level gelatin (*p* < 0.05).

Analysis of the variance of different gelatin levels on the released water (mg H_2_O) sausage showed a significant effect (*p* < 0.05), while storage time had no significant effect (*p* > 0.05), as shown in Figure 2.

The addition of different levels of gelatin affects the binding power of sausage water, which was indicated by the relatively low amount of released water (mg H_2_O) in each gram of sausage when pressed with a filter paper press compared to the 0%. The higher the gelatin level, the less water was released (*p* < 0.05). As shown in Figure 2, there was a significant difference between the 0% and other treatments (*p* < 0.05), but the 2% SFG, 2% BCG, and 3% SFG treatments were not significantly different (*p* > 0.05). The addition of a minimum of 2% SFG has been able to improve the ability of sausages to bind sausage water based on the beef cheek meat, and it seems that the higher the level of gelatin added, the water holding capacity tends to increase. The results of the study using SFG and BCG types also did not show any difference in their water holding capacity. The released water from the sausage that was given the 0% treatment was 38.57 ± 8.01 mg H_2_O/g, greater than the other treatments which were 33.48 ± 5.86 mg H_2_O/g (1% SFG), 27.28 ± 8.42 mg H_2_O/g (2% SFG), 24.06 ± 7.57 mg H_2_O/g (2% BCG), and 23.03 ± 4.98 mg H_2_O/g (3% SFG).

Storage time did not affect the ability of sausages to bind water (*p* > 0.05). The ability of sausages to release water was relatively stable. The amount of released water during storage up to 28 days was between 28.03 ± 7.10 and 29.68 ± 10.65 mg H_2_O/g sausage. The higher the gelatin level, the less the sausage's ability to release water, or in other words the higher the water holding capacity. This was in line with the research of Jridi et al. [14] in turkey sausage which showed an increase in water holding capacity with increasing gelatin levels. Darmanto et al. [47] stated that the ability to bind water is an indicator of protein quality; the higher the ability of the material to bind water, the better the protein quality.

The processing process, especially grinding, causes damage to the myofibril protein structure; as a result, the myofibril properties that can retain meat water are reduced. The addition of gelatin can replace the function of myofibril proteins to resist the release of water and at the same time bind the fat added to the dough to produce sausages with the ability to bind water that can be maintained up to 28 days of storage. This was indicated by the percentage of released water (mg/g) in the sausage which tended to be lower than the 0% (Figure 2). Jridi et al. [14] explained that the addition of gelatin resulted in cross-linking of the chain gelatin covalently to form a matrix that swelled due to the water-containing environment. The water in the form of a gel is then retained with the protein in the dough as a function of the hydrophilic amino acids of gelatin and remains as it was when it was stored at cold temperatures.

### 3.7. pH of Sausage

**Figure 3:**
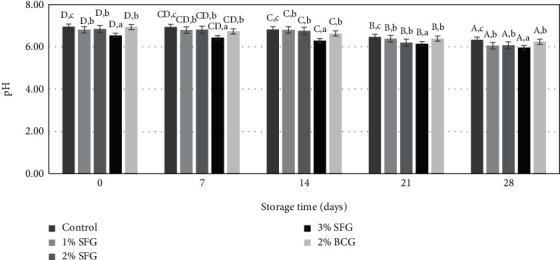
Effect of addition of gelatin to pH of sausage during storage. Different uppercase letters indicate differences in storage time (*p* < 0.05), and lowercase letters indicate differences with level gelatin (*p* < 0.05).

The addition of different gelatin levels affected the pH of sausage (*p* < 0.05), as well as storage time, causing a change in the pH of gelatin (*p* < 0.05), and there was no interaction between gelatin level and storage time on pH (*p* > 0.05). The effect of increasing gelatin level and storage time on sausage pH is presented in Figure 3. The addition of gelatin levels between 1% SFG, 2% SFG, and 2% BCG showed no significant difference (*p* > 0.05). The 0% treatment showed a higher sausage pH, while the additional 3% SFG level resulted in sausages with a lower pH.

The difference in sausage pH between treatments was influenced by different gelatin levels. The addition of gelatin levels, both SFG and BCG, resulted in relatively the same pH range, while the pH of the 3% SFG treatment resulted in sausages with a relatively lower pH among all treatments. This difference in pH may have something to do with the pH of the gelatin used. Sausage 0% treatment showed a relatively higher pH range until storage for 28 days compared to other treatments. According to Ch'ng et al. [11], this has something to do with the accumulation of metabolites caused by microbial action on proteins and amino acids during storage.

### 3.8. Antioxidant Activity in Sausage

**Figure 4:**
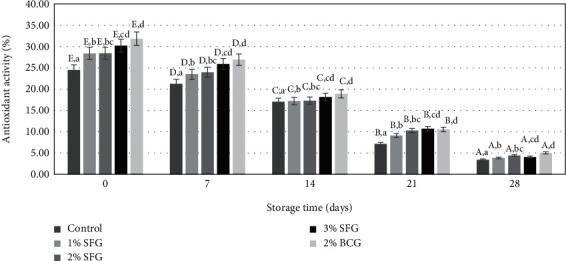
Addition of gelatin level and storage time on antioxidant activity (%) sausage. Different uppercase letters indicate differences in storage time (*p* < 0.05), and lowercase letters indicate differences with level gelatin (*p* < 0.05).

Based on the analysis of variance, both gelatin level and storage time showed a significant effect (*p* < 0.05) on the antioxidant activity of gelatin in sausage meat byproduct, but there was no interaction between the two (*p* > 0.05).  Figure 4 shows the effect of increasing gelatin levels and storage time on antioxidant activity in sausages.

The addition of different gelatin levels showed that there was a difference in antioxidant activity between treatments (*p* < 0.05). The percentage of antioxidant activity in the 0% was the lowest among the treatments, while the addition of 2% BCG level showed the highest values. The addition of 1% SFG and 2% SFG levels was not significantly different, as were 2% SFG and 3% SFG, and between 3% SFG and 2% BCG (*p* > 0.05). The greater the level of SFG added, the antioxidant activity of sausages increased, and the addition of 2% BCG tended to show better activity than the 2% SFG treatment. This indication shows that different types of gelatin can produce different responses to their ability to scavenge free radicals, as well as the antioxidant activity of three types of gelatin from different fish sources, reported by Alemán et al. [48] on flying squid (*Dosidicus gigas*), tuna (*Thunnus* spp.), and halibut (*Hypoglossus* spp.) and Kittiphattanabawon et al. [49] on skin gelatin of *Chiloscyllium punctatum* and *Carcharhinus limbatus*.

The higher the gelatin concentration in the sausage, the greater the antioxidant action. This has something to do with the increased ability of sausages to retain water and fat due to the increased gelatin content. The occurrence of water-protein-fat binding mediated by gelatin produces a compact sausage structure, where water, protein (meat fiber), and fat were trapped between the gelatin solution resulting in a stable bond in a three-dimensional water-protein-fat matrix. The strength of the bonds formed can inhibit the formation of free radicals because of the presence of water or fat that moves freely. The formation of water-protein-fat bonds formed as a barrier to the formation of free radicals due to the oxidation of several fat or protein molecules. The mediation carried out by gelatin cannot be separated from the function of hydrophilic and hydrophobic amino acids.

So far, the use of gelatin as a natural antioxidant was almost not found, but the observations of Sae-leaw et al. [50] in testing the effectiveness of gelatin hydrolysate with different concentrations using the DPPH assay showed a positive correlation of hydrolyzed gelatin concentration with its effectiveness in counteracting free radicals. Nikoo et al. [51] explained that the amino acid peptide sequence in gelatin works to inhibit the mobilization of water molecules into different compartments, and this condition can cause water to remain in a stable state so that the oxidation process that can involve proteins and fats that may occur in a fast time can be extended.

The activity of gelatin as an antioxidant cannot be separated from the function of amino acids, which according to Matsui et al. [52] contributed by the active chain, while according to Jiang et al. [53] was determined by its amino acid sequence. Chi et al. [54] confirmed that the smaller molecular size, the presence of hydrophobic and aromatic amino acid residues, and the amino acid sequence are the key factors that determine the antioxidant activity of proteins, hydrolysates, and peptides. These amino acids react with free radicals and convert them into stable products. It is not known for sure the type of amino acids in SFG and BCG that have the potential as antioxidants. However, it is possible that almost all amino acids can play a role in the process of inhibiting the work of free radicals in sausages as a consequence of oxidation and processing. Lobo et al. [55] highlighted two mechanisms of the working principle of antioxidants, namely, the occurrence of chain breakdown so that reactive amino acids of gelatin will donate electrons to free radicals and antioxidant action directs its effect by donating electrons, chelating metal ions, or acting as co-antioxidants.

Gelatin can work as an antioxidant, although its effectiveness in counteracting free radicals is relatively low compared to hydrolyzed gelatin [49]. Some combinations of amino acids in the peptide sequence are responsible for the gelatin's antioxidant properties. The function of gelatin as an antioxidant is shown by some combinations of amino acids in the peptide sequence. Glycine and proline are two amino acid peptides that can scavenge free radicals, whereas leucine is an amino acid with very strong antioxidant activity [51, 53]. Liu et al. [56] consider tyrosine, tryptophan, and phenylalanine as free radical scavengers. Perhaps further research on the potential of snakehead fish gelatin hydrolysate and the amino acid peptide sequence formed concerning its antioxidant performance and activity can support the above statement.

The longer the storage period, the lower the percentage of antioxidant activity. The storage process can lead to oxidative reactions [57]. Storage for 28 days showed that there was a difference in the antioxidant activity of sausages in counteracting free radicals (*p* < 0.05). This can be seen from the decrease in antioxidant activity every seven days of observation as the length of storage increases. The decrease in antioxidant activity of gelatin in sausages during cold storage was not different from that which occurred in meatballs given pomegranate extract, and it seems that cold storage reduces antioxidant activity more quickly than freezing temperatures [57].

### 3.9. TBA Reactivity of Sausage

**Figure 5:**
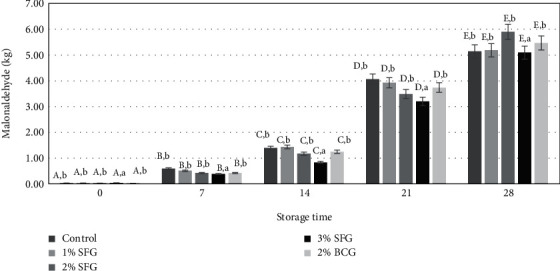
Addition of level gelatin (%) and storage time (days) on TBA reactivity (mg/kg malonaldehyde) sausage. Different uppercase letters indicate differences in storage time (*p* < 0.05), and lowercase letters indicate differences with level gelatin (*p* < 0.05).

Gelatin level and storage time showed in Figure 5 a significant effect (*p* < 0.05) on TBA reactivity of beef cheek-based sausage, but there was no significant interaction between them (*p* > 0.05). The addition of 3% gelatin resulted in relatively lower TBA reactivity, namely, 1.91 mg/kg malonaldehyde compared to other treatments (*p* < 0.05), while the 0% treatment with the addition of 2% SFG and 2% BCG did not show a significant difference (*p* > 0 0.05) with a range of 2.18 to 2.25 mg/kg malonaldehyde.

Gelatin that functions as a binder is a source of amino acids, especially hydrophobic amino acids such as alanine, isoleucine, proline, leucine, phenylalanine, tyrosine, and tryptophan [56]; they act as hydrogen donors through their reactive sites for free radicals resulting in lipid oxidation that can be slowed down [50]. The higher the gelatin content, the fat oxidation indicated by the lower level of TBA reactivity formed, which was expressed as mg malonaldehyde/kg sausage. Storage can trigger fat oxidation. Observations on samples stored at 4 ± 2°C increased the levels of TBA reactivity with storage time (*p* < 0.05). Oxidation was the main cause of damage to meat products [58, 59], during the processes of preparation, processing, and storage due to the depletion of endogenous antioxidants [11, 58]. Lipid oxidation that occurs as a consequence of refrigerated storage causes the formation of lipid peroxidation in the presence of free radicals and subsequently decomposes into malonaldehyde. The addition of the gelatin level helps to maintain the shelf life of the sausage until the rate was slowed down.

In proximate analysis, beef cheek meat can be categorized as lean meat [5]. The processing of beef cheek meat into sausages resulted in sausages containing a certain amount of fat (Table 2). The fat contained in sausages was not chemically stable fat, the addition of other ingredients (nonmeat ingredients) including ice water and processing factors to storage are critical factors for changes in the structure and compartments of sausages, especially oxidation. The instability of sausage products due to some damage by oxidation has implications for a decrease in the shelf life, acceptability, and nutrition of sausages [58]. It seems that the use of gelatin was not only potential as an additive that functions to improve sausage characteristics but was also able to effectively act as an antioxidant that can relatively maintain shelf life. Although not as effective as hydrolyzed gelatin, the inclusion of gelatin in sausage products successfully prevents oxidation-related lipid and possibly protein degradation [49]. The usage of natural antioxidants may enhance the quality of meat products as well as be taken into account for health benefits.

## 4. Conclusion

Gelatin can be replaced with a byproduct of the snakehead fish industry for producing sausages. Snakehead fish gelatin has the potential to serve as a binder and functionally enhance sausage performance, which is determined by the consistency of the emulsion, the amount of cooking loss, the chemical composition, and the texture profile. By confirming scanning electron microscopy, sausage characteristics with the addition of 2% snakehead fish skin gelatin produced sausages with characteristics resembling commercial gelatin. The ability of snakehead fish gelatin to function as a functional binder can increase the ability of sausages to hold water, but it has not been proven to be effective in preventing fat oxidation, which raises levels of malonaldehyde and is characterized by antioxidant activity that tends to decline with cold storage. Gelatin made from fish processing byproducts is a potential substitute for gelatin derived from mammals.

## Figures and Tables

**Table 1:** Emulsion stability (ES) and cooking loss (CL) with the addition of different gelatin levels.

ES (%)	0%	1% SFG	2% SFG	3% SFG	2% BCG
S	16.30 ± 2.92^c^	11.43 ± 4.68^bc^	8.66 ± 3.21^ab^	5.73 ± 0.88^a^	8.81 ± 0.44^ab^
RW	11.53 ± 0.50^b^	7.13 ± 2.73^ab^	5.12 ± 1.72^a^	4.83 ± 1.21^a^	5.68 ± 2.01^ab^
FR	4.77 ± 0.02^b^	4.30 ± 0.06^b^	3.53 ± 0.03^b^	0.90 ± 0.01^a^	3.13 ± 0.02^b^
CL (%)	12.71 ± 3.18^b^	6.67 ± 0.87^a^	6.64 ± 1.08^a^	3.87 ± 2.35^a^	5.87 ± 0.35^a^

^a, b, c^Symbols on the same line indicate significant differences (*p* < 0.05). Mean ± Stdev (*n* = 3). SFG = snakehead fish skin gelatin; BCG = commercial gelatin sourced from bovine bone; S = supernatant; RW = released water; FR = fat released.

**Table 2:** Proximate composition of sausages emulsion with the addition of different gelatin levels.

Composition (%)	0%	1% SFG	2% SFG	3% SFG	2% BCG
Moisture	60.43 ± 1.73^a^	62.09 ± 0.93^ab^	63.27 ± 0.72^b^	63.95 ± 1.07^b^	62.46 ± 1.15^a**b**^
Protein	17.48 ± 0.78^a^	18.84 ± 1.93^a^	18.84 ± 1.04^a^	19.01 ± 0.67^a^	18.69 ± 0.85^a^
Fat	2.41 ± 0.36^a^	2.50 ± 0.29^a^	2.59 ± 0.16^a^	2.60 ± 0.19^a^	2.57 ± 0.15^a^
Ash	2.06 ± 0.38^a^	2.07 ± 0.12^a^	1.97 ± 0.24^a^	1.86 ± 0.27^a^	2.07 ± 0.18^a^

^a, b, c^Symbols on the same line indicate significant differences (*p* < 0.05). Mean ± Stdev (*n* = 3). SFG = snakehead fish skin gelatin; BCG = commercial gelatin sourced from bovine bone.

**Table 3:** Sausage texture profile using different gelatin level addition.

Texture	Hardness (g force)	Cohesive	Elasticity (%)	Shear force (g force)
0%	3012.90 ± 146.89^a^	0.41 ± 0.00^a^	75.87 ± 4.45^a^	10556.67 ± 1268.51^b^
1% SFG	3356.04 ± 645.98^a^	0.43 ± 0.03^a^	76.22 ± 5.18^a^	6833.00 ± 2022.28^a^
2% SFG	3457.70 ± 429.90^a^	0.43 ± 0.10^a^	81.14 ± 1.77^ab^	5899.00 ± 1000.75^a^
3% SFG	3795.10 ± 320.29^ab^	0.43 ± 0.04^a^	87.33 ± 2.16^b^	4747.67 ± 1264.16^a^
2% BCG	4302.70 ± 495.25^b^	0.60 ± 0.06^b^	85.96 ± 7.52^b^	5473.00 ± 493.21^a^

SFG = snakehead fish skin gelatin; BCG = commercial gelatin sourced from bovine bone. ^a, b, c^The mean ± Stdev (*n* = 3) in the same column shows a significant difference (*p* < 0.05).

## Data Availability

Data used and/or analyzed in the study are available from the corresponding author on reasonable request.

## References

[1] Hassoun A., Aït-Kaddour A., Abu-Mahfouz A. M. (2022). The fourth industrial revolution in the food industry—part I: Industry 4.0 technologies. *Critical Reviews in Food Science and Nutrition*.

[2] Kumar V., Muzaddadi A. U., Mann S., Balakrishnan R., Bembem K., Kalnar Y. (2018). Utilization of fish processing waste: a waste to wealth approach. *Compendium of ICAR Summer School Emerging Post-Harvest Engineering and Technological Interventions for Enhancing Farmer’s Income*.

[3] Ranken M. D. (2000). *Hand Book of Meat Product Technology*.

[4] Pereira J., Zhou G., Zhang W. (2016). Effects of rice flour on emulsion stability, organoleptic characteristics and thermal rheology of emulsified sausage. *Journal of Food and Nutrition Research*.

[5] Rosmawati R., Said M. I., Abustam E., Tawali A. B. (2020). Komposisi kimia dan korelasi beberapa karakteristik daging pipi sapi bali. *Jurnal Peternakan Indonesia (Indonesian Journal of Animal Science)*.

[6] Liu D., Nikoo M., Boran G., Zhou P., Regenstein J. M. (2015). Collagen and gelatin. *Annual Review of Food Science and Technology*.

[7] Nurkhoeriyati T., Huda N., Ahmad R. (2011). Gelation properties of spent duck meat surimi-like material produced using acid–alkaline solubilization methods. *Journal of Food Science*.

[8] Nurilmala M., Suryamarevita H., Hizbullah H., Jacoeb A. M., Ochiai Y. (2022). Fish skin as a biomaterial for halal collagen and gelatin. *Saudi Journal of Biological Sciences*.

[9] Vela G., Va M., Ramı A. (2009). Effects of adding fish gelatin on Alaska pollock surimi gels. *Food Hydrocolloids*.

[10] Huda N., Seow E. K., Normawati M. N., Nik Aisyah N. M., Fazilah A., Easa A. M. (2013). Effect of duck feet collagen addition on physicochemical properties of surimi. *International Food Research Journal*.

[11] Ch’ng S. E., Ng M. D., Pindi W., Kang O. L., Abdullah A., Babji A. S. (2014). Chicken sausages formulated with gelatin from different sources: a comparison of sensory acceptability and storage stability. *World Applied Sciences Journal*.

[12] Huda N., Alistar T. L. J., Lim H. W., Nopianti R. (2012). Some quality characteristics of Malaysian commercial fish sausage. *Pakistan Journal of Nutrition*.

[13] Ham Y. K., Song D. H., Noh S. W. (2020). Effects of gelatin hydrolysates addition on technological properties and lipid oxidation of cooked sausage. *Food Science of Animal Resources*.

[14] Jridi M., Nasri R., Salem R. B. (2015). Chemical and biophysical properties of gelatins extracted from the skin of octopus (*Octopus vulgaris*). *LWT- Food Science and Technology*.

[15] Normah I., Syuhadah M. Z. N. (2019). Comparative study on the physicochemical characteristics of chicken sausage incorporated with sutchi catfish (*Pangasius hypophthalmus*) gelatin, carrageenan and pectin. *Food Research*.

[16] Rosmawati, Tawali A. B., Said M. I., Zzaman W., Kobun R., Huda N. (2021). Characteristics of gelatin from skin and bone of snakehead (*Channa striata*) extracted with different temperature and time. *Potravinarstvo Slovak Journal of Food Sciences*.

[17] Meliana E., Agustini T. W., Kurniasih R. A. (2022). The effect of kappa carrageenan addition on the emulsion stability of milkfish (*Chanos chanos*) sausage. *Saintek Perikanan: Indonesian Journal of Fisheries Science and Technology*.

[18] Tan C. C., Karim A. A., Uthumporn U., Ghazali F. C. (2020). Effect extraction temperature on the emulsifying properties of gelatin from black tilapia (*Oreochromis mossambicus*) skin. *Food Hydrocolloids*.

[19] Mohan A. (2014). *Basics of Sausage Making, Formulation, Processing & Safety*.

[20] Mohamed H. M., Emara M. M., Nouman T. M. (2016). Effect of cooking temperatures on characteristics and microstructure of camel meat emulsion sausages. *Journal of the Science of Food and Agriculture*.

[21] AOAC (2006). *Official Methods of Analysis of AOAC International*.

[22] Asmaa A. A., Zzaman W., Yang T. A. (2015). Effect of superheated steam cooking on fat and fatty acid composition of chicken sausage. *International Food Research Journal*.

[23] Muthia D., Nurul H., Noryati I. (2010). The effects of tapioca, wheat, sago and potato flours on the physicochemical and sensory properties of duck sausage. *International Food Research Journal*.

[24] Rombe G. S., Tahir M. M., Tawali A. B. (2020). Physicochemical characteristics of pempek premix flour made from mackerel fish (*Scomberomoru scommersoni*) surimipowder. *IOP Conference Series: Earth and Environmental Science*.

[25] Tarladgis B. G., Watts B. M., Younathan N. T., Dugan L. (1960). A distillation method for the quantitative determination of malonaldehyde in rancid foods. *Journal of the American Oil Chemists' Society*.

[26] Blois M. S. (1958). Antioxidant determinations by the use of a stable free radical. *Nature*.

[27] Barbut S. (1995). Importance of fat emulsification and protein matrix characteristics in meat batter stability. *Journal of Muscle Foods*.

[28] Santhi D., Kalaikannan A., Sureshkumar S. (2017). Factors influencing meat emulsion properties and product texture: a review. *Critical Reviews in Food Science and Nutrition*.

[29] Feiner G. (2006). *Meat Products Handbook*.

[30] Ayadi A., Kechaou A., Makni I., Attia H. (2009). Influence of carrageenan addition on Turkey meat sausages properties. *Journal of Food Engineering*.

[31] Lee C. H., Chin K. B. (2016). Effects of pork gelatin levels on the physicochemical and textural properties of model sausages at different fat levels. *LWT- Food Science and Technology*.

[32] Souissi N., Jridi M., Nasri R. (2015). Effects of the edible cuttlefish gelatin on textural, sensorial and physicochemical quality of octopus sausage. *LWT- Food Science and Technology*.

[33] Yeo E. J., Kim H. W., Hwang K. E. (2014). Effect of duck feet gelatin concentration on physicochemical, textural, and sensory properties of duck meat jellies. *Korean Journal for Food Science of Animal Resources*.

[34] Cheng Q., Sun D. W. (2008). Factors affecting the water holding capacity of red meat products: a review of recent research advances. *Critical Reviews in Food Science and Nutrition*.

[35] Pietrasik Z. (1999). Effect of content of protein, fat and modified starch on binding textural characteristics, and colour of comminuted scalded sausages. *Meat Science*.

[36] Schilling M. W., Mink L. E., Gochenour P. S., Marriott N. G., Alvarado C. Z. (2003). Utilization of pork collagen for functionality improvement of boneless cured ham manufactured from pale, soft, and exudative pork. *Meat Science*.

[37] Samatra M. Y., Mohd Noor N. Q. I., Razali U. H. M., Bakar J., Shaarani S. M. (2022). Bovidae-based gelatin: extractions method, physicochemical and functional properties, applications, and future trends. *Comprehensive Reviews in Food Science and Food Safety*.

[38] Cofrades S., Antoniou I., Solas M. T., Herrero A. M., Jiménez-Colmenero F. (2013). Preparation and impact of multiple (water-in-oil-in-water) emulsions in meat systems. *Food Chemistry*.

[39] Chorbadzhiev P., Zsivanovits G., Gradinarska D., Danov K., Valkova-Jorgova K. (2017). Improvement of texture profile attributes of cooked sausage type “Krenvirsh”. *Bulgarian Journal of Agricultural Science*.

[40] Santana P., Huda N., Yang T. A. (2013). The addition of hydrocolloids (carboxymethylcellulose, alginate and konjac) to improve the physicochemical properties and sensory characteristics of fish sausage formulated with surimi powder. *Turkish Journal of Fisheries and Aquatic Sciences*.

[41] Singh Y., Blaisdell J. L., Herum F. L., Stevens K., Cahill V. (1985). Texture profile parameters of cooked frankfurter emulsions as influenced by cooking treatment. *Journal of Texture Studies*.

[42] Resurreccion A. V. A. (2004). Sensory aspects of consumer choices for meat and meat products. *Meat Science*.

[43] Sousa S. C., Fragoso S. P., Penna C. R. A. (2017). Quality parameters of frankfurter-type sausages with partial replacement of fat by hydrolyzed collagen. *LWT- Food Science and Technology*.

[44] Prestes R. C., Carneiro E. B. B., Demiate I. M. (2012). Hydrolyzed collagen, modified starch and guar gum addition in Turkey ham. *Ciência Rural*.

[45] Parés D., Pèlach M. A., Toldrà M., Saguer E., Tarrés Q., Carretero C. (2018). Nanofibrillated cellulose as functional ingredient in emulsion-type meat products. *Food and Bioprocess Technology*.

[46] Qi W., Wu J., Shu Y. (2020). Microstructure and physiochemical properties of meat sausages based on nanocellulose-stabilized emulsions. *International Journal of Biological Macromolecules*.

[47] Darmanto Y. S., Agustini T. W., Swastawati F. (2012). Effect of various fish bone collagens on the quality of myofibril fish protein during dehydration process. *Teknologi dan Industri Pangan*.

[48] Alemán A., Giménez B., Montero P., Gómez-Guillén M. C. (2011). Antioxidant activity of several marine skin gelatins. *LWT- Food Science and Technology*.

[49] Kittiphattanabawon P., Benjakul S., Visessanguan W., Shahidi F. (2012). Gelatin hydrolysate from blacktip shark skin prepared using papaya latex enzyme: antioxidant activity and its potential in model systems. *Food Chemistry*.

[50] Sae-leaw T., Callaghan Y. C. O., Benjakul S., Brien N. M. O. (2016). Antioxidant activities and selected characteristics of gelatin hydrolysates from seabass (*Lates calcarifer*) skin as affected by production processes. *Journal of Food Science and Technology Technology*.

[51] Nikoo M., Benjakul S., Ehsani A. (2014). Antioxidant and cryoprotective effects of a tetrapeptide isolated from Amur sturgeon skin gelatin. *Journal of Functional Foods*.

[52] Matsui R., Honda R., Kanome M. (2018). Designing antioxidant peptides based on the antioxidant properties of the amino acid side-chains. *Food Chemistry*.

[53] Jiang Y., Zhang M., Lin S., Cheng S. (2018). Contribution of specific amino acid and secondary structure to the antioxidant property of corn gluten proteins. *Food Research International*.

[54] Chi C., Hu F., Wang B., Li Z., Luo H. (2015). Influence of amino acid compositions and peptide profiles on antioxidant capacities of two protein hydrolysates from skipjack tuna (*Katsuwonus pelamis*) dark muscle. *Marine Drugs*.

[55] Lobo V., Patil A., Phatak A., Chandra N. (2010). Free radicals, antioxidants and functional foods: impact on human health. *Pharmacognosy Reviews*.

[56] Liu R., Xing L., Fu Q., Zhou G., Zhang W. (2016). A review of antioxidant peptides derived from meat muscle and by-products. *Antioxidants*.

[57] Turgut S. S., Soyer A., Işıkçı F. (2016). Effect of pomegranate peel extract on lipid and protein oxidation in beef meatballs during refrigerated storage. *Meat Science*.

[58] Falowo A. B., Fayemi P. O., Muchenje V. (2014). Natural antioxidants against lipid – protein oxidative deterioration in meat and meat products: a review. *Food Research International*.

[59] Gheisari H. R. (2009). Correlation between acid, TBA, peroxide and iodine values, catalase and glutathione peroxidase activities of chicken, cattle and camel meat during refrigerated storage. *Veterinary World*.

